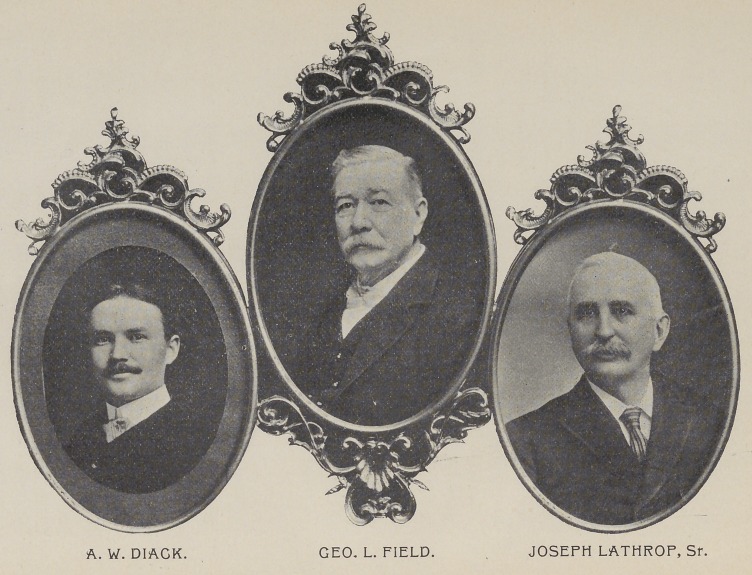# Editorial

**Published:** 1901-05-15

**Authors:** 


### EDITORIAL.

### THE OHIO, MICHIGAN AND INDIANA TRI-STATE MEETING.

June 4th, 5th and 6th, at Indianapolis, will be held the third triennial convocation of the dentists of Ohio, Michigan and Indiana. In some respects these meetings have been the most remarkable gathering of dentists in the history of the profession. The things that have specially characterized them have been the large attendance, the value of the work accomplished, and the fraternal spirit engendered.

To Mr. C. S. Bigelow, of the Ransom & Randolph Co., of Toledo, belongs the credit of first suggesting such a union meeting. Owing to his persistently following up his suggestion, the Michigan, Ohio and Indiana State Societies appointed committees to confer on the subject. A joint meeting of these committees was held at Old Point Comfort, Va., August 6, 1894, and arrangements were then completed for holding the first meeting at Detroit June 18, 19 and 20, 1895. A general committee, consisting of Dr. L. L. Barber, of Ohio; Dr. J. Ward House, of Michigan, and Dr. Geo. E. Hunt, of Indiana, was appointed at that time to make arrangements for the program, etc. A local committee of arrangements and entertainment was raised, consisting of Drs. Geo. L. Field, Joseph Lathrop, Sr., and A. W. Diack, of Detroit. It was hoped that there might be an attendance of three hundred. Every one was agreeably surprised, however, when the count showed nearly five hundred and fifty were present. The sessions of the meeting were held in the admirably appointed buildings of the Detroit College of Medicine, and consisted of four literary sessions and two half-day clinics. The social features were completed in a boat ride on the Detroit River to the St. Clair Flats where a royal fish dinner was served. Those present will not soon forget this trip, with the visit to the establishment of Parke, Davis & Co., the music and other incidents of the trip. The transactions of this meeting were published in the *Dental Cosmos* of 1895, and also in separate pamphlet form.

The wonderful success of this undertaking opened the way for the second meeting, which, on the invitation of the Ohio State Dental Society, was held three years later at Put-in-Bay, Lake Erie, at the "Hotel Victory." The charming surroundings of this place attracted a large attendance, and over six hundred were present on this occasion. This large attendance made it possible to present a most attractive literary program. The clinics were not, however, numerous, because of the impracticability of giving them for lack of facilities. The social features here were all that could be desired. At great expense the Ohio Society brought from Cincinnati the famous "Apollo Club" of nearly one hundred fine men singers, and their singing was greatly enjoyed. This meeting was held June 21, 22, 23, 1898, and the proceedings were published in the *Dental Cosmos* for 1898 and 1899, and also in pamphlet form. The entire committee of the Ohio Society did all that was possible to make this meeting a success from every standpoint. Dr. J. R. Callahan, the chairmain of the committee, was largely responsible for the complete arrangements made for the comfort and entertainment of the guests.

The Indiana meeting promises to eclipse all the others; we trust it may not be "three times and out." Drs. Geo. E. Hunt, J. R. Callahan and J. Ward House are the members of the general committee, and they promise a program that will claim attention, and be worth any one's while to go miles to hear and see, for it will not be all papers and discussion, but there will be clinics and exhibits of all up-to-date methods and appliances. These meetings are a kind of free-for-all, and any reputable dentist, whether a member of the State Society or not, is welcome and will be shown every courtesy.

The Indiana dentists are making every effort to furnish social entertainment that will engender fraternal relations. The meetings will be held in a large club room which is capable of accommodating in every way a large number of people. And the hotel accommodations are unsurpassed. It is announced that the Indiana ladies will also do a little entertaining on their own account. So that wives, sisters and sweethearts will be taken care of in an entirely satisfactory manner. The committee is planning for an attendance of eight hundred. If their expectations are realized, and each one should bring an offering and leave it on the altar of dental science, what a grand addition would be made to the resources of our profession! After all, is this not the motive and object of all such concerted enterprises? None of us know it all; and there is no one but can learn something from others if he will. What is still more to the point, there is no one of any considerable experience but has encountered some peculiarity which if made known and put on record would be available for comparative study or application in the solution of some of the complex problems which hinder the perfecting of dental science or art.

It is safe to say that there has been no such chance in years for the dentists of this part of the country to attend so really helpful a meeting as this promises, and it may be a long time before a similar privilege shall offer. Low railroad and hotel fares can be obtained, and everything that can be done to make the trip easy will be done. So make your plans to attend this meeting, leave the babies with grandmother and bring the wife along. A complete program can be obtained, if you have not yet seen it, by addressing Dr. Geo. E. Hunt, 131 E. Ohio Street, Indianapolis.

THE Dental Hospital oi London Medical School opens its session of 1901 May 1st in a new building in Leicester square, which is doubtless the best building of its kind in the old world. It should be, as the building, ground and equipment are said to have cost not far from $350,000. It is encumbered with a mortgage of about $270,000 and has annual charges of over $18,000 to meet. It however has some sources of income from rentals. It would seem that such a financial handicap would not be conducive to the highest educational values. The building is said to be admirably equipped with modern facilities. Our English cousins wake up slowly, but in this case they seem to be thoroughly aroused. We wish the new venture every possible success.

J. WARD HOUSE.

C. E. HUNT.

J. R. CALLAHAN.

A. W. DIACK.

GEO. L. FIELD.

JOSEPH LATHROP, Sr.

## Figures and Tables

**Figure f1:**
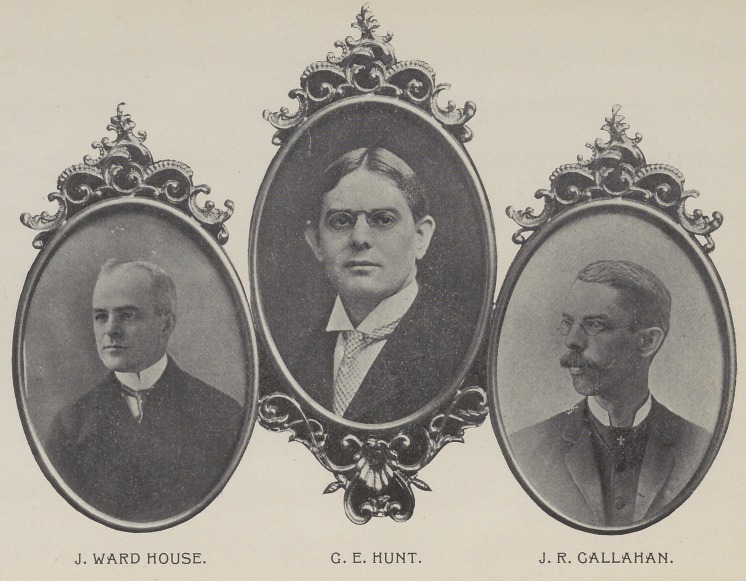


**Figure f2:**